# Challenges of scaling-up of TB-HIV integrated service delivery in Ghana

**DOI:** 10.1371/journal.pone.0235843

**Published:** 2020-07-09

**Authors:** Prince Justin Anku, Joshua Amo-Adjei, David Doku, Akwasi Kumi-Kyereme

**Affiliations:** Department of Population and Health, University of Cape Coast, Cape Coast, Ghana; Liverpool School of Tropical Medicine, UNITED KINGDOM

## Abstract

Integration of tuberculosis and HIV services in many resource-limited settings, including Ghana, has been far from optimal despite the existence of policy frameworks for integration. A previous study among programme managers and other stakeholders at the national level has documented tardiness in committing to the integration of services. In this paper, we aimed at unravelling pertinent challenges that confront TB-HIV integrated service delivery. Data were obtained from interviews with 31 individual health care providers operating under different models of TB-HIV service delivery. The study is framed around the Complexity Theory. We applied inductive and deductive techniques to code the data and validations were done through inter-rater mechanisms. The analysis was done with the assistance of QSR NVivo version 12. We found evidence of a convivial working relationship between TB-HIV service providers at the facility level. However, the interactions vary across models of care–the lesser the level of integration, the lesser the complexities for interactions that ensued. This had resulted in operational challenges on account of how the two-disease environment interacts with the other components of the health system. These challenges included; weak/inappropriate infrastructure, frail coordination between the two programmes and hospital administrators, under-staffing in comprehensive TB–HIV management, use of community facility under the Directly-Observed Treatment (DOT) protocols, and financial constraints. To fully appropriate the enormous benefits of TB-HIV service integration, there is a need to address these challenges.

## Introduction

Tuberculosis (TB) remains a leading cause of mortality and ill-health among people living with Human Immunodeficiency Virus (HIV), especially in resource-limited countries, even though significant progress has been made in the fight against the two epidemics over the years [1]. Indeed, the emergence of HIV in sub-Saharan Africa also increased substantially the incidence of TB in the region [2]. There is substantial evidence regarding the linkages between HIV and TB, with a higher likelihood of mortality among co-infected clients [3,4]. Despite the high risk of HIV clientsdeveloping TB, programmes at the global, national, and local levels started largely with a vertical approach with little or no coordination [5]. This has resulted in the poorly coordinated management of the syndemic, with deleterious effects on clients and operational difficulties for service providers, especially in resource-limited settings [6].

As a response to the unabated TB-HIV co-morbidity, the World Health Organization (WHO) proposed TB-HIV service integration at least at the facility level [7]. There is, however, no consensus with regards to the form (whether partial or full) of integration and the levels at which integration should occur [8]. As a result, various models (linkage, collaboration, full integration) have been implemented with several challenges across various settings [9]. For instance, in a linkage model, when a patient is diagnosed with either of the two infections, he/she is referred to another facility or unit to be tested for the other. The collaborated model is concerned with the partial integration of services whereby a person who has been diagnosed through TB services will also be counselled and tested for HIV and then referred if positive. In a fully integrated model of care, all services for TB and HIV are provided in a single facility and by the same service providers. An overwhelming body of evidence, however, suggests that the fully integrated option offers optimum benefits for clients, health systems and workers [10,11].

In many resource-limited settings like Ghana, achieving widespread integration of TB-HIV care is still unsatisfactory [12], regardless of a documented intent towards full integration [13]. While this is not the first empirical discourse on TB-HIV integration in the country, this, to the best of our knowledge, marks the first attempt to investigate operational challenges of TB-HIV integration from the perspectives of service providers at the facility level. Our study was guided and discussed within the remit of Complexity Theory [14].

## Theoretical framework: The complexity theory

Complexity theory in healthcare is concerned with functionality and changes within a given healthcare system, beginning from the assumption that the field of healthcare delivery has become increasingly complex [15,16]. Complexity has been applied across various disciplines with each discipline offering a unique understanding of the term. Manson divided complexity theory into three; *algorithmic complexity*, *deterministic complexity*, and *aggregate complexity* [14]. In this paper, we subscribed to “aggregate complexity” which concerns the relationships between individual components in a complex adaptive system (in this case, integrated TB-HIV care operating within a wider health care system). Aggregate complexity is generally regarded as the qualitative component of the complexity theory [16].

Complexity theory views healthcare facilities organized around specific chronic disease management as “complex adaptive systems”. This complex adaptive system is a group of individual agents, specialized health workers in the field of TB and HIV management, whose actions are interconnected [17] and the interactions among the specialized units are more critical than the discrete actions of individual components [18]. According to Mason [14], understanding aggregate complexity will require an exploration of key sets of interrelated concepts that define a complex system. These key concepts include: relationships between entities; internal structures and the surrounding environment; learning and emergent behaviour, and the various ways by which a complex system can transform and improve [15].

A cornerstone of aggregate complexity is the *relationships* that exist among the various components. Here, the concern is about the interactions among the various components of the system; TB and HIV service providers, the TB/chest unit, HIV/counselling unit, laboratory, X-ray unit, pharmacy/dispensary, and other units/wards with a direct or indirect relationship with TB and HIV management and the clients. With regards to the *internal structure*, it is assumed that the components of a system and their relationships are not different. Consequently, the actions of the related units are or should aim at ensuring a better outcome for health service users/clients, especially the most vulnerable. Thus, in the various models of care; separate, linkage, collaboration, and fully integrated systems, each unit and their staff have to follow the laid down treatment protocols. A key element in the existence of a complex system is the *environment*. Here, the environment is the broader political, social, and organizational context—the physical infrastructure (units) for the management of TB and HIV in the health facility. To a large extent, the environment determines the level of interactions that will take place between the components of a complex system.

However, a complex system like healthcare is not entirely dependent on the environment, but actively shapes and reacts to changes over time because of the range of suitable internal mechanism [19,20]. In rare cases where the components lack the ability to respond to new interactions with the environment, there is a potential for catastrophic results. In a public healthcare system where unpredictability is high, there is a need for changes to be made over time. A critical characteristic of a complex system is *self-organization*, which allows for changes to the internal structure to better interact with the environment. Unlike many organizational theories that assume stability, a major strength of aggregate complexity lies with its position that systems such as healthcare are constantly changing their internal structure and external environment. The complexity theory offered us an opportunity to understand how a complex system like integrated TB-HIV care operates, obstacles to scaling-up integration and the need for individual units to interact in order to produce the desired result.

## Study context

The need to ensure effective management of TB in Ghana saw the establishment of the National TB Control Programme (NTP) in 1994. Since then, TB management and related activities in Ghana have been under the auspices of the NTP. Currently, the management of HIV and AIDS in Ghana is under two State-supported institutions; the Ghana AIDS Commission (GAC) which is a supra-ministerial and multi-sectoral body established by Act 613, 2002 of parliament, under the chairmanship of the President of the Republic of Ghana and the National AIDS Control Programme (NACP) [21].

Both antiretroviral therapy (ART) and TB treatment in Ghana started largely with a vertical and centralized approach with very few health facilities providing services. However, both have since been decentralized to other health facilities across the country, resulting in 1057 TB treatment centres and 160 ART clinics by the end of 2012 [22]. Until 2007, TB and HIV management in Ghana were carried out vertically, with referral services the only means of integration. Following the WHOs recommendation for closer integration of TB and HIV programmes [7], the Ghana Health Service in collaboration with the NACP and NTP developed a policy guideline on the clinical as well as the general management of TB-HIV co-infection. TB-HIV service integration is expected to have taken place in all healthcare facilities in Ghana by 2015 [13].

## Materials and methods

Data for the study were drawn from a total of 31 service providers– 11 institutional TB Coordinators, 10 institutional HIV Coordinators, five Medical Officers, three supporting nursing staffs, a Pharmacist and a Laboratory Technician from 12 selected health facilities across four regions of Ghana. The participants were purposively selected based on their direct involvement as well as long working experience in the management of TB and or HIV in the selected facilities. The four regions and the 12 facilities (three from each region) were also purposively selected due to the relatively high burden of TB and HIV in their catchment areas. In each of the selected regions, either the regional hospital or a Teaching Hospital that serves as the highest level of clinical referrals was purposively selected in addition to two other facilities. However, in the region with the highest prevalence of TB-HIV, the major health facility in the district with the highest prevalence of TB-HIV was purposively selected in addition to the regional hospital and one other facility.

Permission for the study to be conducted was obtained from the management of each of the selected facilities. The purpose and nature of the study were explained to the study participants and written consent was obtained individually from each participant. Given the focus of the study, a highly flexible semi-structured interview guide was developed and used for the data collection [23]. The interviews were based on one-on-one interaction with the participants and were all tape-recorded with their consents. This was done to ensure that we capture reality in the exact words of the individual participants [24]. The first author conducted all the interviews and transcribed all the recorded interviews. At the end of each day of data collection, we took note of both old and newly emerging issues in the data through playback of the recorded interviews. This was done to keep track of the issues in the data in order to identify issues that will require further probing in the subsequent interviews. By the 31st interview, we observed that we have had enough “Information Power” based on the established criteria [25]. On average, each interview lasted 45 minutes. Ethical approval for the study was obtained from the Ghana Health Service Ethical Review Committee (ERC) (GHS-ERC: 15/10/15) in Accra, Ghana.

The data were analysed at two levels following both inductive and deductive coding approaches. At the first level, we employed an inductive coding technique to explore a range of codes from the data. These codes were analysed and put into themes, sub-themes and categories. The emerged themes and categories were further analysed for commonalities, variations and disagreement [26]. At the second level, we followed a deductive coding approach where we adequately explored themes and categories that fell under the key constructs of the complexity theory [27]. This analytical strategy–inductive before deductive coding was adopted to avoid imposing the theoretical framework on the data and allowed themes to emerge freely. All analyses were done with the assistance of QSR NVivo 12 software. In order to ensure the reliability and validity of our findings, we employed an inter-rater coding technique where the first and second authors independently coded the data at both levels. The other authors reviewed the codes and all authors discussed the codes and the themes and categories that were subsequently developed. Our findings are presented and discussed within the remit of the Complexity Theory. We followed the proposed steps put forward by Kannampallil et al, [15]. Even though our focus is to understand the challenges to TB-HIV service integration, we also, first of all, explored issues relating to interactions between service providers of both programmes as well as the internal structure for TB and HIV management in accordance with the theory.

## Results

### Background characteristics of study participants

A total of 31 service providers were interviewed in the selected health facilities across the four selected regions in Ghana. The participants were between the ages of 26 and 59 years with varying levels of experiences in TB and or HIV management. All the participants have at least three years of working experience in management of TB and or HIV with the most experienced service provider having 30 years of experience in TB and HIV management. The participants occupied a wide range of positions in the management of TB and HIV in the selected facilities across the four selected regions in Ghana. They were made up of 36 percent Institutional TB Coordinators, 32 percent Institutional HIV coordinators, 16 percent Medical Officers, 3 percent Pharmacist, 10 percent Nurse/Counsellor, and 3 percent Laboratory Technician. The participants were drawn from various levels of health facilities including; Teaching Hospital, Regional Hospitals, Metropolitan Hospitals, Sub-Metro Hospitals, Municipal Hospitals, District Hospitals and Sub-District Hospitals across four regions of Ghana with various models of TB-HIV service delivery (separate, linkage, collaboration, and full integration) under practice. The participants were affiliated to either the National AIDS Control Programme (NACP) or National TB Control Programme (NTP) or both programmes in the case of facilities that are practising full integration model. The summary of the background characteristics of the study participants is presented in Table 1.

**Table 1:** Background characteristics of study participants.

Background characteristic	Frequency	Percentage (%)
**Service Providers**		
**Sex**		
Male	14	45.2
Female	17	54.8
**Age**		
20–29 years	7	22.6
30–39 years	11	35.4
40–49 years	6	19.4
50–59 years	7	22.6
**Programme Affiliation**		
NTP	12	38.7
NACP	13	41.9
NTP/NACP	6	19.4
**Position**		
TB Coordinator	11	35.5
HIV Coordinator	10	32.3
Medical Officer	5	16.1
Pharmacist	1	3.2
Counsellor/Nurse	3	9.7
Lab. Technician	1	3.2
**Years of Work Experience**		
1–9 years	24	77.4
10–19 years	4	12.9
20–29 years	2	6.5
30–39 years	1	3.2

Fieldwork, 2019.

### Relationships/Interactions

Generally, the participants shared the view that there is a cordial working relationship between service providers of the two programmes or units. This convivial relationship was observed to be at strongest among workers in fully integrated models of care. The participants disclosed that they work as a team and are very satisfied with the working relationships that exist among their respective teams since all of them can provide both TB and HIV care to clients. It was evident that the strong working relationship among service providers in full integration model emanated from the fact that both the TB and HIV units were under the Public Health Department of such health facilities.

*We work together as a team*. *The units are not separated*; *everything is done here in this building*. *We work together very well*. *In fact*, *when a team member is not around*, *anybody within the team can perform his/her work*. *We usually go to workshops together as a team so it is more of teamwork*.

(55-year-old Facility HIV coordinator, working under full integration model)

Even though service providers in other models of care also disclosed that they have a cordial working relationship, it was not as strong as what was observed in full integration model. This was evident in the accusation and counter-accusation among service providers for TB and HIV at the two units. The following quote reflects this observation:

*I may say we* [service providers for TB] *have a cordial relationship with them* [HIV-unit]. *Sometimes*, *we do have little quarrels pertaining to report writing–they have to write a report to us about their* [clients], *the number of them that are coughing*, *the number of new* [clients] *that they have diagnosed as HIV–positive* … *It is mandatory for us TB-HIV team to have periodic meetings at the facility level*, *but sometimes on a meeting day*, *you will wait for them*, *but no one will turn up*. *But aside that*, *I may say*, *our relationship is cordial*.

(31-year-old Institutional TB coordinator, been working with the programme for 5 years,—a collaborative model of care)

*Sometimes*, *they are too concerned with their part* [TB treatment] *… They will forget to continuously remind* [clients] *with both TB and HIV that*, *even after TB treatment is completed*, *they still need to continue their HIV treatment* … *Because*, *the units are not together*, *from time to time we have little disagreements*. *But*, *generally*, *we have a good working relationship*.

(31-year-old Nurse/Counsellor, been working with the HIV programme for 3 years,—collaborative model)

### Internal structure

**Fig 1:**
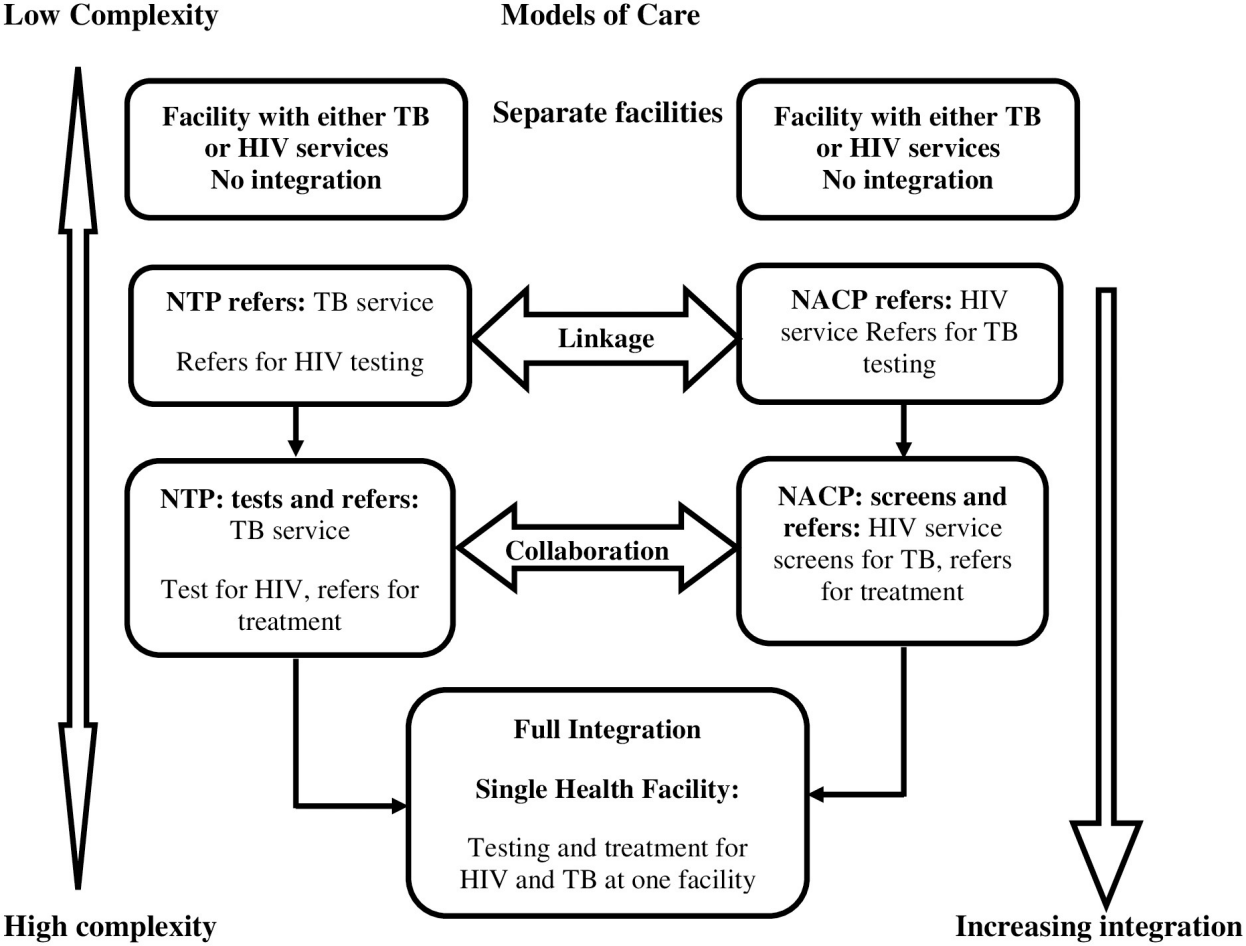
Models of care and levels of complexity.

We observed a complex interaction between and among components (service providers and clients) across the various models of care–the lesser the level of integration, the lesser the complexities in the system of care (see, Fig 1). Put differently, the level of complexity increases as we move from a separate model (no integration) to linkage, to collaboration and finally to full integration model of care. There is a laydown protocol to be followed by service providers in TB and HIV management. This protocol expects service providers for the respective diseases to constantly interact with each other. As such, when separate facilities or different units of the same facility provide service, which is structurally located away from each other, the system becomes less complex with attendant challenges.

*In this facility* [VCT unit], *all HIV positive clients are usually screened for TB*. *After the screening*, *based on the responses*, *we refer the person to the lab to produce sputum for testing* … *If positive*, *we then refer the person to the Chest Clinic to have treatment*. *Similarly*, *when a TB client is detected*, *those at the Chest Clinic also tests for HIV and if positive*, *they refer the client to our unit for management*. (58 years old HIV coordinator, 15 years of working experience—Collaboration Model) The complexity is increased when services are provided in a fully integrated system (Fig 1).

*Every TB client that we receive is screened for HIV and if negative*, *we continue with the treatment*, *but if positive*, *we refer the client to the HIV clinic for management*. *Luckily for us*, *both units are at the same place so we just send the person to the next room to go and see the HIV coordinator* … *We*, *first of all*, *start with the treatment of the TB and then later introduce the HIV treatment*, *depending on the patient*.

(26 years old Disease Control Officer/TB coordinator, 3 years working experience—Full Integration Model)

### Environment

The interactions/relationships among the various components, coupled with the internal structure in the management of TB and HIV will require that services are adequately integrated. However, we found several challenges, which are preventing services from being adequately integrated. These obstacles are largely a reflection of the environment of TB-HIV service delivery. Among the key issues we noted from the analysis were: inappropriate infrastructure, weak engagements among programme staff and hospitals’ management and inadequate staff in TB and HIV management.

#### Inappropriate infrastructure and potential stigma

Infrastructure was found to be one of the major challenges to TB-HIV service integration. In most of the facilities where there appears to be some level of collaboration between the HIV unit and the chest clinic/ TB unit, participants were of the view that their efforts to effectively collaborate are often undermined by the physical distance between the two units. They accentuated that TB and HIV services need to be fully integrated in order to deliver the best possible care to the clients. However, in facilities where the two units were not together, it is more difficult to follow the right protocol in the management of the two diseases. Aside from the physical distance between the two units posing a challenge, issues regarding stigma and privacy were also raised as hindering service integration in most of the facilities. The lack of appropriate infrastructure to provide TB-HIV services was expressed by almost all service providers delivering services in separate, linkage and collaborative systems.

*Inadequate* [inappropriate] *infrastructure is our major problem in this facility*. *Housing the two units* [TB and HIV units] *at one place will help the system to improve*. *This is very necessary for effective service delivery*, *but as it stands now*, *we have the HIV unit as part of the Public Health Unit and the TB Unit is separated up there*. *Ideally*, *we are supposed to be working very closely*, *but we are not able to do so because of the* [physical infrastructural] *distance between the two Units* … *We must do it* [integrate] *in such a way that we don’t end up opening ourselves and the* [clients] *to stigma*.

(39 years old Institutional HIV coordinator, 8 years working experience–a collaborative model of care)

#### Weak engagements among programme staff and hospitals’ management

Unsteady coupling between top managers of NTP, NACP and management of the various hospitals also emerged as a major challenge to TB-HIV service integration. The participants revealed that, even though clients could access treatment and care for both TB and HIV at the same facility, the two units are too far apart from each other. This often poses challenges to both service providers and clients. The hospital authorities and programme managers are, however, not showing enough willingness to commit resources towards full integration of services. As such, service providers are not able to effectively collaborate to deliver the best possible care to clients. Aside from diagnostic services, there seems to be no much collaboration when it comes to treatment and data management. This is what one of the participants said when asked to comment on the possibility of full integration at the facility:

*Yes*, *it is possible*! *But our managers and hospital authorities always complain there is no money*. *It is possible to do it because it is being done at other facilities* … *Both TB and HIV services are provided at the Public Health Unit*. *Here*, *the two units are far from each other and sometimes when you send* [clients]*to the other unit*, *they don’t go—they only turn around and go home*.

(31-year-old service provider, working as TB coordinator for 5 years–Collaborative model of care)

#### Inadequate staff in TB and HIV management

Inadequate staff was one of the major concerns raised by the participants regarding TB-HIV management. Most of the participants shared the view that there are very limited health personnel, especially Medical Officers who are involved in the management of TB and HIV at the facility level. In both units (VCT and TB units), the participants emphasized shortage of staffs and increase workload as a challenge to the integration of services since they are expected to provide both TB and HIV counselling services, diagnosis, data management, follow-up on clients, embark on community outreach programmes, and the general management of the syndemic. As such, taking up extra duties due to integration does not appeal to them very well. Most of the participants shared the view that health personnel, especially medical doctors are often reluctant to work in the area of TB and HIV management. In addition, some participants were concerned that full integration of services may lead to the reassignment of some providers to other units, thereby increasing the workload on the few service providers.

*We need more prescribers—the doctors*! *As it stands now*, *we have only one doctor who is willing to help in this area*. *For the other doctors*, *I don’t really know whether it’s because of stigma or something*. *It looks like they are feeling reluctant to work with us and you know*, *this is the biggest hospital in the region and it serves as a referral centre so we tend to get a lot of* [clients] … *Only a few nurses are involved in TB and HIV management*. *As if TB-HIV management is reserved for just a few of us who have taken it upon ourselves to work with the programme*. *That is why in some facilities*, *people* [service providers] *are not willing to integrate*.

(45-year-old Institutional HIV coordinator with 6 years working experience–Full integrated model of care)

#### Directly-Observed Treatment (DOT) strategy in TB management

The DOT strategy was also seen as posing a challenge to clients receiving care in the integrated system. Even though service providers acknowledged the importance of co-infected clients receiving care for both TB and HIV at the same facility, they are sometimes left incapable of resolving the situation where clients have to use separate facilities for TB and HIV care. Under the DOT strategy, clients are required to receive treatment and care at a facility in their communities or one that is closer to their communities. Whereas TB services are available in nearly all health facilities, including Health Centres in Ghana, HIV services are somewhat limited to major health facilities across the country. Therefore, if service providers have to strictly go by the DOT strategy, clients will have to use a facility that is closer to their place of residence for the TB treatment and then another facility for HIV services where it is not available in the facility that is providing the TB services. This concern was shared by several participants and this was what one has to say:

…*TB* [management] *is supposed to be DOT* [Directly-Observed Treatment], *so the clients are supposed to take their TB drugs* [medications] *as close to their homes as possible*. *Yet*, *HIV medicines are not available at the facilities in smaller communities*. *So*, *what it means is that we have people here who have to receive their TB drugs in a small community somewhere*, *but still have to come to us for the HIV services*. *So*, *it makes it such that it is difficult to have a one-stop-shop for them* [co-infected clients] … *The best would have been that they come to one place* [and] *they get their HIV and TB drugs*.

(49-year-old Medical Officer/Institutional TB-HIV coordinator with 10 years working experience–Full integrated model of care)

#### Funding constraints

Funding issues also emerged from the data as posing challenges to the general activities of the two disease control programmes, as well as the integration of services. Whereas the participants acknowledged the tremendous contribution made by donor agencies, especially the Global Fund, they also expressed concerns about the ever-dwindling of the funds. In recent times, some donor agencies like the Global Fund have resorted to giving out funds for joint activities of TB and HIV control, with malaria being added in some instances. Such initiatives by the international organizations have reinforced the need to integrate services, especially at the facility level which will result in resource pooling. However, the participants accentuated that, to adequately integrate services, there is the need to invest in suitable infrastructure which will enhance collaboration between the two units. The two control programmes rely heavily on donor funding, but the donors often do not provide funds for infrastructure and are usually “activity-specific” in terms of the funds. The following quote highlights this concern shared by the respondents:

*The issue is that the* [control] *programmes* [NACP and NTP] *rely heavily on donor funding to carry out our activities*. *Now that the funds are no longer coming like before*, *we are increasingly facing so many challenges* … *Another major challenge is infrastructure; where we can provide both TB-HIV services*. *This will require money*, *but the funds are no longer coming and even if they come*, *the donors are specific and often they are not concerned about funding infrastructure*. *The hospital authorities are also reluctant in this regard*. *So*, *it makes it difficult to adequately integrate services*.

(48 years old Institutional HIV coordinator with 9 years working experience–a collaborative model of care)

## Discussion

This study seeks to unravel the pertinent challenges that confront TB-HIV integrated service delivery using the complexity theory as a framework. Following the recommendation for TB-HIV service integration by the World Health Organization, several resource-limited countries have implemented this strategy (albeit various models), which has largely proven to have a positive impact on the management of the syndemic [28,29].

It is, however, worth mentioning that the success or otherwise of TB-HIV service integration to ensure effective management of the syndemic depends largely on the service providers and the level of coordination that exist between them [30]. Our study revealed a generally cordial working relationship between service providers for TB and HIV, especially in fully integrated models of care. This cordial relationship is critical to ensuring the delivery of high-quality integrated care. As posits by the complexity theory, this cordial relationship between the components (service providers for TB and HIV) is essential for the ultimate survival of the system [20].

Concerning the internal structure, our study revealed a growing complex interaction between and among service providers for TB and HIV and co-infected clients. This complexity was higher in fully integrated models of care as compared to a partial integration. Plsek and Greenhalgh [19] noted that as a system becomes more and more complex, there is the likelihood of non-linearity and the system adapts better to situations. Evidence suggests that in less complex systems of TB-HIV care, referrals for co-infected clients to access comprehensive care is a major challenge and at the same time, missed opportunities for HIV clients to get enrolled on ART exist [31]. These challenges can, however, be addressed through service delivery in a better integrated (more complex) system [28]. There is evidence to show that full integration of TB-HIV services is considered most appropriate and acceptable among clients, especially co-infected clients [32].

Stigma within the health sector and the general public is a major challenge in the fight against TB and HIV in Ghana [33,34]. With stigma, an important factor, any effort in the management of TB and HIV (two infectious diseases, which carry stigma) must take into consideration the protection of clients against stigma. Institutional challenges relating to a lack of appropriate infrastructure in implementing TB-HIV collaborative services have been documented [35]. The management of TB and HIV in most resource-limited settings started with the vertical approach with two different control programmes (e.g. National AIDS Control Programme and National Tuberculosis Control Programme in Ghana). With such a vertical approach, most health facilities designated different units (often far apart from each other) to the management of TB and HIV. Integration of services requires either bringing the two units together or reasonably close to each other and ensure effective collaboration [7]. This is where some facilities are faced with challenges since integration was not initially considered in setting up of the two units [36]. Within the framework of the complexity theory, the health facilities can manage and deliver TB-HIV services to clients in the face of the infrastructural challenges because a complex system is not totally dependent on the environment, but actively shapes and reacts to changes because of the suitable internal components [19]. A critical component in this instance are the dedicated TB and HIV services providers at the various health facilities. However, as posited by the theory where a component of the system lacks the ability to respond to the environment, there is the likelihood of catastrophic results.

Our study also revealed weak engagements on the part of programme managers at the national level and hospital authorities as a major challenge to TB-HIV service integration. Policy document by the Ghana Health Service entreats all health facilities to integrate TB and HIV services by the end of 2015 [13]. However, TB and HIV service integration in Ghana is far from optimum, even though some progress has been made in recent years. A previous study among programme managers for both TB and HIV revealed some level of reluctance to commit to the integration of services due to various reasons, including the potential for leadership crises and increased workload [12]. In some sub-Saharan Africa countries where there is evidence of inadequate infection control protocol, there is a reluctance to commit to TB-HIV integration due to concerns about nosocomial infection [37,35].

Of particular note are the challenges of inadequate staff and the DOT strategy in TB management hindering TB-HIV service integration. This finding is consistent with results from a study conducted in South Africa, where it was found that inadequate health staffs with a special interest in TB and HIV care, especially in rural settings as a major hindrance to TB-HIV service integration [38]. DOT strategy has been widely used in TB management to help address treatment default and non-adherence [39]. However, the challenge here is that, with co-infectedclients, ART is not available in every facility across the country. Whereas it is easy for the clients to receive care for TB even at a health centre within the community, ART is not available in such facilities. As such, service integration becomes very difficult. While there is the need for clients to receive care at facilities that are closer to them as much as possible under DOT [40], efforts should also be made to decentralize ART to primary health facilities as it has been proven to be feasible [19]. Whiles DOT remains a widely used strategy, its relevance in the current TB management and control has been questioned [41]. The finding from this study adds to the growing call for a review of the DOT strategy in TB management.

Our study also revealed funding constraints as a barrier to service integration as well as generally having negative effects on the activities of both control programmes. Funding constraints for TB and HIV control activities in Ghana due to over-reliance on donors have been documented [42,36]. The volatile nature of disease-specific funding due to priority setting and changing politics on the part of the donors makes overreliance on such funds risky [43]. Investment in infrastructure, which according to the participants is critical in ensuring integration of services, has unfortunately not been a priority for donor organizations. With the current situation where donors are continuously reducing funds for the programmes, government and various health institutions must show more commitment by looking at alternative means of funding activities for TB and HIV control programmes while at the same time addressing the barriers to TB-HIV service integration.

Notwithstanding the meaningful insight provided by this study regarding the challenges to TB-HIV service integration, there is an inherent limitation. Only 12 health facilities from four regions of Ghana were included in this study and this limits the extent to which the results can be generalized. However, our goal was to understand the phenomenon rather than to generalise. Besides, the strong theoretical basis for the study and a large number of study participants added to the strength of this study. As such, the results can be transferred across similar contexts.

## Conclusion

The complexity theory provided us with the basis to understand the challenges with regards to the scaling-up of TB-HIV integrated service delivery in Ghana. This study revealed a number of challenges to TB-HIV service integration, which largely emanated from the environment of care within the complex system. These include; inappropriate infrastructure and potential stigma, weak engagements among programme managers and hospital authorities, inadequate staff in TB and HIV management, DOT strategy in TB management, and funding constraints. With evidence pointing to the viability of full integration of services and its positive impact on the management of TB and HIV in many resource-limited settings, there is the need for all stakeholders to address the challenges to service integration. Full integration of service will enhance the complexity of the system, which can have a positive impact on early diagnosis for both infections, improve interactions between service providers for both control programmes and clients, enhance information sharing and improve the general management of the syndemic.

## Supporting information

S1 Appendix (DOCX)

## References

[1] World Health Organization. Global tuberculosis report. Geneva: 2019 https://www.who.int/tb/publications/global_report/en/

[2] Getahun H, Gunneberg C, Granich R, Nunn P. HIV infection-associated tuberculosis: the epidemiology and the response. Clinical Infectious Disease. 2010; 50(3): S201–07. 10.1086/651492 20397949

[3] Silva Escada RO, Velasque L, Ribeiro SR, Cardoso SW, Spindola Marins LM, Grinsztejn E, et al Mortality in patients with HIV-1 and tuberculosis co-infection in Rio de Janeiro, Brazil—associated factors and causes of death. BMC Infectious Diseases. 2017; 17(373). 10.1186/s12879-017-2473-y PMC5450415 28558689

[4] Rossetto M, Brand EM, Rodrigues RM, Serrant L, Teixeira LB. Factors associated with hospitalization and death among TB/HIV co-infected persons in Porto Alegre, Brazil. PLoS One. 2019; 14(1): e0209174 10.1371/journal.pone.0209174 30601842 PMC6314623

[5] Friedland G, Harries A, Coetzee D. Implementation issues in tuberculosis/HIV program collaboration and integration: 3 case studies. Journal of Infectious Disease. 2007; 196(1): S114–23. 10.1086/518664 17624820

[6] Harries AD, Zachariah R, Corbett EL, Lawn SD, Santos-Filho ET, Chimzizi R, et al The HIV-associated tuberculosis epidemic—when will we act? Lancet. 2010; 375: 1906–19. 10.1016/S0140-6736(10)60409-6 20488516

[7] WHO. WHO Policy on Collaborative TB/HIV Activities: Guidelines for National Programmes and Other Stakeholders. Geneva; 2012. 23586124

[8] Legido-Quigley L, Montgomery CM, Khan P, Atun R, Fakoya A, Getahun H, et al Integrating tuberculosis and HIV services in low- and- middle-income countries: a systematic review. Tropical Medicine and International Health. 2013; 18(2): 199–211. 10.1111/tmi.12029 23217030

[9] WHO. World Health Organization: Global Tuberculosis Report 2012. Geneva; 2012.

[10] Kerschberger B, Hilderbrand K, Boulle AM, Coetzee D, Goemaere E, De Azevedo V, et al The Effect of Complete Integration of HIV and TB Services on Time to Initiation of Antiretroviral Therapy: A Before-After Study. PLoS One. 2012; 7(10): e46988 10.1371/journal.pone.0046988 23071690 PMC3465310

[11] Schulz SA, Draper HR, Naidoo P. A comparative study of tuberculosis patients initiated on ART and receiving different models of TB-HIV care. International Journal of Tuberculosis and Lung Diseases. 2013; 17(12): 1558–1563 10.5588/ijtld.13.0247 24200268

[12] Amo-Adjei J, Kumi-Kyereme A, Amo HF, Awusabo-Asare K. The politics of tuberculosis and HIV service integration in Ghana. Social Science & Medicine. 2014; 117: 42–49. 25042543 10.1016/j.socscimed.2014.07.008

[13] Ghana Health Service, USAID-Ghana, QHP. Implementation of TB/HIV Collaborative Activities in Ghana: Technical Policy and Guidelines. Accra: 2007.

[14] Manson S. Simplifying complexity: a review of complexity theory. Geoform. 2001; 32(2): 405–414

[15] Kannampallil TG, Schauer GF, Cohen T, Patel VL. Considering complexity in healthcare systems. Journal of Biomedical Informatics. 2011; 44(6): 943–947. 10.1016/j.jbi.2011.06.006 21763459

[16] Kernick D. Complexity and Healthcare Organization: A view from the street Oxford, UK: Radcliffe Publishing; 2004.

[17] Hanson WR, Ford R. Complexity leadership in healthcare: Leader network awareness. Procedia—Social and Behavioural Sciences. 2010; 2(4): 6587–6596.

[18] Weberg D. Complexity leadership: a healthcare imperative. Nursing Forum. 2012; 47(4): 268–277. 10.1111/j.1744-6198.2012.00276.x 23127241

[19] Plsek PE, Greenhalgh T. Complexity Science: The challenge of complexity in Health Care. British Medical Journal. 2001; 323: 625–28. 10.1136/bmj.323.7313.625 11557716 PMC1121189

[20] Belrhiti Z, Giralt AN, Marchal B. Complex leadership in healthcare: A scooping review. International Journal of Health Policy and Management. 2018; 7(12): 1073–1084. 10.15171/ijhpm.2018.75 30709082 PMC6358662

[21] Ghana AIDS Commission. Ghana AIDS Commission web site. [Online].; 2019 [Accessed 2020 March 27. https://www.ghanaids.gov.gh/pages/about-us

[22] Ghana Health Services. 2011 Annual Report. Accra; 2012.

[23] McIntosh M, Morse JM. Situating and constructing diversity in semi-structured interviews. Global Qualitative Nursing Research. 2015; 1–12. 10.1177/2333393615597674 PMC5342650 28462313

[24] Nordstrom SN. Not So Innocent Anymore: Making Recording Devices Matter in Qualitative Interviews. Qualitative Inquiry. 2015; 21(4): 388–401

[25] Malterud K, Siersma VD, Guassora AD. Sample Size in Qualitative Interview Studies: Guided by Information Power. Qualitative Health Research. 2015; 26(13): 1753–1760. 10.1177/1049732315617444 26613970

[26] Morse JM. Analytic Strategies and Sample Size. Qualitative Health Research. 2015; 25(10): 1317–1318. 10.1177/1049732315602867 26355022

[27] MacFarlane A, O'Reilly-de Brun M. Using a Theory-Driven Conceptual Framework in Qualitative Health Research. Qualitative Health Research. 2012; 22(5): 607–618. 10.1177/1049732311431898 22203386

[28] Owiti P, Zachariah R, Bissell K, Kumar AM, Diero L, Carter EJ, et al Integrating tuberculosis and HIV services in rural Kenya: uptake and outcomes. Public Health Action. 2015; 5(1): 36–44. 10.5588/pha.14.0092 26400600 PMC4525370

[29] Pathmanathan I, Pasipamire M, Pals S, Dokubo EK, Preko P, Ao T, et al High uptake of antiretroviral therapy among HIV-positive TB patients receiving co-located services in Swaziland. PLoS One. 2018; 13(5): e0196831 10.1371/journal.pone.0196831 29768503 PMC5955520

[30] Kumwenda M, Tom S, Chan AK, Mwinjiwa E, Sodhi S, Joshua M, et al Reasons for accepting or refusing HIV services among tuberculosis patients at a TB-HIV integration clinic in Malawi. International Journal of Tuberculosis and Lung Disease. 2011; 15(2): 1663–1668. 22118175 10.5588/ijtld.10.0741

[31] Miyano S, Muvuma S, Ishikawa N, Hiroyoshi E, Msiska C, Syakantu G. Healthcare provision for HIV co-infected tuberculosis patients in rural Zambia: an observational cohort study at primary care centers. BMC Health Services Research. 2013; 13(397). 10.1186/1472-6963-13-397 PMC3851769 24103082

[32] Anku PJ, Amo-Adjei J, Doku DT, Kumi-Kyereme A. Integration of tuberculosis and HIV services: Exploring the perspectives of co-infected patients in Ghana. Global Public Health. 2018: 10.1080/17441692.2017.1385823 28984493

[33] Dodor EA, Kelly S. Manifestation of tuberculosis stigma within the healthcare system: the case of Sekondi-Takoradi Metropolitan district in Ghana. Health Policy. 2010; 98(2): 195–202. 20637520 10.1016/j.healthpol.2010.06.017

[34] Amo-Adjei J, Awusabo-Asare K. Reflections on tuberculosis diagnosis and treatment outcomes in Ghana. Archives of Public Health. 2013; 70(1): 10.1186/2049-3258-71-22 23971675 PMC3765431

[35] Kalonji D, Mahomed OH. Health system challenges affecting HIV and tuberculosis integration at primary healthcare clinics in Durban, South Africa. African Journal of Primary Health Care & Family Medicine. 2019; 11(1): a1831. 10.4102/phcfm.v11i1.1831 PMC6556920 31170790

[36] Amo-Adjei J. Views of health service providers on obstacles to tuberculosis control in Ghana. Infectious Disease of Poverty. 2013; 2(9). 10.1186/2049-9957-2-9 PMC3710189 23849141

[37] Gandhi NR, Moll AP, Lalloo U, Pawinski R, Zeller K, Moodley P, et al Successful integration of tuberculosis and HIV treatment in rural South Africa: the Sizonqoba study. Journal of Acquired Immune Deficiency Syndrome. 2009; 50(1): 37–43. 10.1097/QAI.0b013e31818ce6c4 19295333

[38] Nansera D, Bajunirwe F, Kabakyenga J, Asiimwe PK, Mayanja-Kizza H. Opportunities and barriers for implementation of integrated TB and HIV care in lower level health units: experiences from rural western Ugandan district. African Health Sciences. 2010; 10(4): 312–319. 21416031 PMC3052807

[39] Karumbi J, Garner P. Directly observed therapy for treating tuberculosis. Cochrane Database of Systematic. 2015; 2015(5): CD003343. 10.1002/14651858.CD003343.pub4 PMC4460720 26022367

[40] De Lima VY, Evans D, Page-Shipp L, Barnard A, Sanne I, Menezes CN, et al Linkage to Care and Treatment for TB and HIV among People Newly Diagnosed with TB or HIV-Associated TB at a Large, Inner City South African Hospital. PLoS One. 2013; 8(1): e49140 10.1371/journal.pone.0049140 23341869 PMC3547004

[41] McLaren ZM, Milliken AA, Meyer AJ, Sharp AR. Does directly observed therapy improve tuberculosis treatment? More evidence is needed to guide tuberculosis policy. BMC Infectious Diseases. 2016; 16(537). 10.1186/s12879-016-1862-y PMC5050573 27716104

[42] Adjei S, Nazzar A, Seddoh A, Blok L, Plummer D. The Impact of HIV and AIDS Funding and Programing on Health System Strengthening in Ghana. Amsterdam; 2011.

[43] Jenniskens F, Tiendrebeogo G, Coolen A, Blok L, Kouanda S, Sataru F, et al How countries cope with competing demands and expectations: perspectives of different stakeholders on priority setting and resource allocation for health in the era of HIV and AIDS. BMC Public Health. 2012; 12(1271): 10.1186/1471-2458-12-1071 23231820 PMC3549279

