# Supplementary material for: Challenges of scaling-up of TB-HIV integrated service delivery in Ghana
Source: PLoS One. 2020 Jul 9;15(7):e0235843. doi: 10.1371/journal.pone.0235843 (PMC7347185; doi:10.1371/journal.pone.0235843)
Supplement: S1 Appendix — (DOCX) [file pone.0235843.s001.docx]

APPENDIX D

**UNIVERSITY OF CAPE COAST**

**COLLEGE OF HUMANITIES AND LEGAL STUDIES**

**FACULTY OF SOCIAL SCIENCES**

**DEPARTMENT OF POPULATION AND HEALTH**

**In-Depth Interview Guide for Service Providers**

1. **BACKGROUND INFORMATION**
2. Sex
3. Age................................
4. Type of facility: 1= Regional hospital 2= District Hospital 3= Polyclinic 4= Clinic 5= Other [specify].................................
5. Programme affiliation...........................
6. Current Position.....................................
7. How many years all together have you being working as [ ]
8. Have you ever had any in-service training, seminar or workshop on TB-HIV management? **Probe:**

- When was the last time?
- What were the issues covered?
- Was the training relevant? How relevant?

1. **Barriers/Challenges to TB-HIV service integration**
2. I will like you to tell me about how you carry out service for persons with TB taking into account HIV in this facility.
3. What are the guidelines for delivering services for TB-HIV co-infected patients?

- Probe: Specific roles by NACP and NTP official/facility coordinators and other service providers
- What do you think about the nature of the working relationship between the two units? **(Ask in relation to the team members in case of full integration)**

Probe: Are you satisfied? Please give reasons

1. What can you say about the relevance/importance of providing TB services taking into account HIV?

- Probe but don’t prompt! eg: Early initiation of treatment for both infections, adherence to treatment, reduced loss to follow up. etc

1. Can you please tell me about the current most pressing need of this facility with regards to TB-HIV integrated service delivery?

- Why is it the most pressing need? ***Encourage the participant to provide details.***

1. What are some of the barriers to providing TB services taking into account HIV in this facility? *You may* *probe but don’t prompt!* eg: Inadequate staff, limited capacity (infrastructure) of the facility to provide integrated service, nosocomial infection, funding, conflict between NACP and NTP officials. ***Encourage the participant to provide details.***
2. How do these barriers affect or hinder service delivery?
3. How can these barriers or challenges be overcome?
4. Do you have anything else to say generally about TB and HIV management in Ghana?

**Thanks for your time and cooperation!**
